# Editorial

**Published:** 1861-07

**Authors:** 


### Editorial.

---

THE INFLUENCE OF ALCOHOLIC DRINKS ON THE DEVELOPMENT AND PROGRESS OF PULMONARY TUBERCULOSIS.—At the last annual meeting of the American Medical Association the senior editor of this journal presented to the section on Practical Medicine a paper on the subject, presented in the above caption. The paper was subsequently published in the Transactions of the Association, and a synopsis of it may be found in the February number of the EXAMINER.

The appearance of the paper in the Transactions has elicited brief comments in several Medical journals, some of which are rather curious specimens of criticism.

For instance, the *Cleveland Medical Gazette*, after quoting the conclusions appended to the paper, simply adds: "Dr. DAVIS is an *ultraist* on this question, and we are satisfied the *present* experience and opinions of the mass of the profession do not coincide with those presented by him."

The *American Medical Monthly* commences its reference to the paper as follows: "We do not know that it would be possible to make Dr. DAVIS look with complacency upon any use of alcoholic drinks. From his papers, including with this some upon kindred topics, *we judge that he is an uncompromising teetotaler;* a man very good in his way, but not on that account the most fit person to judge of the effects of alcoholic drinks."

These comments suggest several questions which we would like to have answered. And first, what constitutes "an *ultraist*" and "*an uncompromising teetotaler*"? Is it one who simply avoids all use of alcoholic drinks *as beverages?* If so, we cheerfully plead guilty to the charge.

If anything more than this is meant, then we deny the charge, and demand of its authors their proof. But if refusing to use alcoholic drinks, as beverages, makes a man "an ultraist," we suppose a refusal to keep the mouth full of tobacco would make a man "an ultraist" on that subject. And by the same process of reasoning, a refusal to make *opium* an article of diet would constitute "an ultraist" on that subject.

Again, if a man does not, himself, use any particular article of diet or drink, and is therefore, according to the logic of our respected cotemporaries, "an ultraist," does such ultraism incapacitate him for judging correctly of the effects of such articles upon others? If it does, will our Cleveland and New York cotemporaries inform us *just* how many drinks per week or per day, will be sufficient to remove the charge of *ultraism*, and render us capable of judging correctly concerning the effects of alcohol on others? From what we had seen in our ordinary intercourse with men, we had not supposed that the use of alcoholic drinks either improved the intellectual faculties, or rendered the judgment more reliable. Neither had we discovered that an *appetite* for those drinks rendered the mental operations any more impartial or correct.

Finally, what has our individual opinions in reference to the propriety or impropriety of the use of alcoholic drinks, as beverages, to do with a simple record of facts in relation to a given number of cases of tuberculosis?

The reviewer in the New York journal, goes so far as to bring up the "Maine Law," and to give us the benefit of his opinion on that statute. Now, we ask again, what has our

"uncompromising teetotalism," or even the "Maine Law" to do with the record of cases of tuberculosis, given in the paper we had the honor to present to the American Medical Association?

Our New York critic complains that the reasoning in our paper was not *logical.* Perhaps the following *syllogism* would be regarded by him as *logically* correct, viz:

Dr. Davis has reported 210 cases of pulmonary tuberculosis and their relation to the use of alcoholic drinks.—

Dr. Davis is an "*uncompromising teetotaller*"—

Therefore, the cases of tuberculosis reported by Dr. Davis are of no value.

In the introduction to our paper, we stated as a reason for recording cases, that the idea had been extensively promulgated that alcoholic beverages, including wine and beer, were capable of preventing or retarding pulmonary tuberculosis. In regard to this, the writer in the *American Medical Monthly* says: "We do not remember to have seen or heard the *idea announced*, which was the cause of the statistical record of our author." Did that writer ever see or hear of a valuable work known as "Wood's Therapeutics and Pharmæcology"?

If he will turn to Vol. 1, page 666, of that work he will find the *idea* very plainly announced in the following paragraph: "Nature, while planting in so large a proportion of the human family a disposition to scrofulous or tuberculous complaints, seems to have provided, in the *fermented liquors*, what, if properly used, may be considered in some degree a *counteracting agent.*"

But Dr. Wood does not restrict the prophylactic influence to "fermented liquors" alone; for on the next page he says, "Physicians have often noticed that drunkards seldom die of phthisis."—And again: "Out of 117 cases of confirmed drunkards, whose bodies were examined after death by Dr. Oysten, there were only two who exhibited any evidence of tuberculous disease of the lungs."

We doubt whether a respectable work, on either materia medica or practical medicine, has been published in this country during the last eight years, that does not clearly announce the idea, that alcoholic drinks are more or less prophylactic against phthisis. And if our New York critic has never seen or heard of that idea, we fear he has given himself more trouble about the *Maine Law*, than about the ordinary text-books of the profession.

Again he says: "Now, does phthisis pulmonalis prevail among them (those who drink wine, beer, or stronger spirits) more than among teetotallers? Do those who are of tuberculous families develop this disease more certainly or more rapidly if they use these beverages, than if they totally abstain?" And then he complains that our conclusions are not "logical answers" to these questions. Certainly they are not; and for the very good reason, that they are not the questions we had under consideration. We did not entertain or discuss the question whether alcoholic beverages were *causes* of phthisis! But exactly the reverse, namely, whether they were *preventives* or prophylactics.

Having carefully recorded 210 cases, and finding that of the whole number, 68 had habitually used these beverages, from one to twelve years before the active symptoms of phthisis were noticed; 91 had used them occasionally; and 51 not at all; and on further examination, finding that among the 68 habitual users of these beverages were men of all classes, from the day laborer to the judge of the higher courts, and of all degrees of freedom in such use, from a single cup of beer at meal time, to such excess as ended in delirium tremens, we very naturally, if not *logically*, drew the inference, "That neither the action of alcohol on the functions of the human system, nor the actual results of experience, furnish any evidence that these stimulants are capable of either retarding or preventing the development of tuberculosis."

If one man drinks a mug of ale at each meal for five years, and at the end of that time is found to have genuine tubercular phthisis—if another drinks wine in a similar manner and with a similar development—and if a third drinks a glass of whisky from one to three times a day for a similar series of years, and yet finds himself laboring under the same disease, what is the legitimate conclusion? Why, simply that these drinks did not prevent the development of the disease. Is there any want of logic in that? If on extending the inquiry we find 50 or 100 similar cases—or quite as many of that character as of those who do not drink any of those beverages, then we may legitimately and logically vary the conclusion, and say that these drinks have *no power* to prevent the development of phthisis. But the reviewer in the *Cleveland Medical Gazette* says: "We are satisfied the *present* experience and opinions of the mass of the profession do not coincide with those presented by him." We do not doubt the correctness of this statement. And it is precisely for that reason that we called the attention of the profession to the cases and inferences contained in our paper. If we had supposed that the conclusions at which we arrived, in reference to the prophylactic powers of alcoholic drinks, were already the generally received opinions of the great mass of the profession, we should have deemed it an unjustifiable waste of time, to have occupied the attention of the Association with the subject. But how long is it since it was in accordance with "the *experience* and opinions of the great mass of the profession" to keep consumptive patients carefully excluded from the open air, and confined in warm rooms with antiphlogistic medicines? How long is it since it coincided with the "experience and opinions" of the profession, to treat almost all the fevers and phlegmasiæ with venesection and antiphlogistics? Can any one say that "the *present* experience and opinions of the mass of the profession" are any more infallible than the *past?* We wish our cotemporaries would give us fewer mere *opinions*, and more carefully recorded facts.

Western Army Medical Matters.—In accordance with a general order from the Secretary of War, Gov. Yates has appointed the following gentlemen an Examining Board for applicants for Surgeoncies and Assistant Surgeoncies to the volunteer forces, mustered into the service of the United States from this State: Prof. H. A. Johnson, M. D., Chicago, President; Drs. Bryan, (Sycamore,) Davis, (Paris,) Roskoten, (Peoria,) and Wing, (Collinsville.) These gentlemen have been in session at Springfield some three weeks, and will probably remain in session until about the 15th inst. On the 8th, we learn they adjourned to Cairo, for examinations at that place, for one week. A letter from the President, to the junior editor, under date of July 2, says: "We have examined twenty-one applicants and passed *seven* to the grade of Surgeon, and, as you will understand, the results of our labor have been to disturb the relations of some who have been heretofore attached to regiments." So far as we learn, the action of the former Board, which was purely a State affair, has been very fully endorsed by the present one, and the allusion in the last sentence of Dr. Johnson's letter, we take to be to a number of independent gentlemen who refused to come before the former Board for examination, but relied for their appointments upon political influence and favoritism.

The rules to be observed in appearing before the present Board, are similar to those of the regular army Boards. Applications must be made to the Governor for permission to appear for examination, which application must be accompanied by respectable testimonials of professional, moral and physical qualifications. Permission being given, the applicant presents his order for examination to the Board, by which it is registered, and in due order, he is summoned for examination; surgeons already attached to regiments having priority over those unattached. The appointments to service are made by the Governor, usually on recommendations of the colonels.

——Owing to the changes, and consequent confusion arising from the above action, we are unable to present, as we had hoped to, anything of particular interest of camp and field practice, from special correspondents, but give what we deem worthy of note from other sources.

Notwithstanding a considerable amount of sickness during the wet season, which prevailed during the early part of the campaign at Cairo, the general health at that point is good. In some positions of unusual exposure there are reported a number of cases of typhoid fever, and one death on the 30th ult. The *Cairo Camp Register* of June 15th, reports 772 cases of sickness, treated at the hospital there, from the 27th day of April, and up to that time, we believe, *no deaths* had occurred, in the hospital, certainly a remarkably favorable exhibit for a locality so much dreaded.

——SUB-COMMITTEE B, of the Sanitary Commission, elsewhere noticed, consisting of the Rev. Dr. Bellows of New York, and Drs. I. S. Newberry of Cleveland, and W. H. Murray of Cincinnati, have made a satisfactory official inspection of the company quarters and regimental hospitals at Camp Defiance, and Dr. Wright, of Cincinnati, Surgeon-in-chief of the Department of the West, (?) accompanied Major-General Mc Clellan on his late visit to the same post—Dr. W. inspecting the hospitals. Eight or ten women-nurses are on duty in the Cairo hospitals, under charge of Mrs. Yates of this city, acting under authority from Miss Dix, and, as we learn, with the happiest results.

——IN THE REGIMENTS stationed at Chicago, there has been an epidemic of measles, and one or two cases of typhoid fever have occurred. There have also been a few surgical cases, one of a gun-shot wound in the face with buck-shot and shingle nails, causing very severe laceration; another of a bayonet-stab in the chest; a private in the Irish Brigade broke his leg by falling from an upper window of the barracks—was removed to the Mercy Hospital; several cases of hernia and contusions also occurred among the German Dragoons—but in all the foregoing cases, recoveries have been made, or are satisfactorily progressing.

ARMY SANITARY COMMISSION.—The President, at the request of the Medical Bureau of the U. S. Army, has appointed Rev. HENRY W. BELLOWS, D. D., President; Prof. A. D. BACHE, Vice-President; ELISHA HARRIS, M. D., Corresponding Secretary; GEO. W. CULLUM, U. S. A., ALEX. E. SHIRAS, U. S. A., ROBERT C. WOOD, M. D., U. S. A., WM. H. VAN BUREN, M. D., WOLCOTT GIBBS, M. D., SAMUEL G. HOWE, M. D., CORNELIUS R. AGNEW, M. D., and J. S. NEWBERRY, M. D., and GEO. T. STRONG, Treasurer, a *Commission of In-*

*quiry and Advice in Respect of the Sanitary Interests of the United States Forces.* This *Commission* and its plan of organization meets the approval of the War Department, and all persons in the employ of the U. S. Government are directed and enjoined to respect and further its objects to the utmost of their ability. The Surgeon-General also issues an order in its behalf. The Plan of Organization, as published, proposes, in brief, to do to the fullest extent what is implied in the title we have given, viz: first, to *inquire* into the theoretical, actual and desirable conditions of the troops; and second, to *advise* with the Government, Medical Bureau and War Department as to what steps are necessary to insure the highest sanitary condition of the army in every point of view; to see to the carrying out the orders, emanating from their counsels, and to advise with State Governments and benevolent associations, to the end of securing harmony of plans and actions, and abundant supplies of money and goods.

If the *Commission* does not fail to accomplish anything, by attemping to do too much, nor fritters away in petty details, time, which should be spent in laying broad, comprehensive plans, with the execution of which the Medical Bureau and the War Department would be naturally charged,—if it avoids these errors, then we cannot but hail this as one of the most notable epochs in the medical history of our times,—since it not only secures the benefits of the most scientific treatment and counsel to the largest and most valuable volunteer army the world ever saw, but it establishes a precedent, in the recognition by government of the value and importance of the Medical Profession, from which good cannot fail to accrue.

PERSONAL.—PROF. FRANK H. HAMILTON, the eminent New York surgeon and author, has been appointed to the 31st Regiment, N. Y. V. M....DR. LUTHER V. BELL, formerly in charge of the McLean Insane Asylum, Mass., has been appointed to the 11th Mass. Regiment....Dr. ELISHA HARRIS has withdrawn from the editorial staff of the *American Medical Times*, in order to devote himself more thoroughly to the duties of the Corresponding Secretaryship of the Army Sanitary Commission....HENRY GRAY, the eminent anatomist, and author of the popular text-book, died recently in London of confluent small-pox, æt. 36....CLEMENT A. FINLEY, M. D., the present Surgeon-General, is a native of Ohio, and has been in the service forty-three years. DR. SATTERLEE, also of Ohio, we believe, is the next oldest Surgeon on the list.

VOLUNTEER WOMEN NURSES.—Miss DOROTHEA L. DIX has been appointed Superintendent of the women nurses, who are hereafter to be supplied to the hospitals, during the war, in obedience to the demands of public sentiment and the humane instincts of the age. Associations are at work in some of the Eastern cities, selecting and training applicants for such service; and some of the Military Hospitals in this State, as elsewhere, have been already supplied. Mrs. YATES, of this city, is the Matron-in-Charge for this State, with her head-quarters at Cairo.

THE SURGEON-GENERAL authorizes the several medical directors of the army, when they have reason to doubt the professional competency of any of the medical officers under their charge, to organize a board of not less than three medical officers, which shall examine said officers of questioned professional capacity, and decide whether they are competent to the performance of their duties. If the decision of the board is adverse, they will cease to be in the military service of the United States.

---

WE REGRET to learn that the issue of Dr. GIBBS' proposed *Year Book of American Contributions to Medical Science and Literature*, is postponed on account of the paucity of subscribers, troublesome times and the pecuniary embarrassments of the masses. The Dr., however, convinced of its utility, announces his readiness to undertake it at any time when there shall appear a hope of its paying expenses; and, meanwhile, proposes preparing a *Summary* of medical journal literature which will enrich the pages of that enterprising publication, the *Phila. Med. and Surg. Reporter.*

Tough Lunar Caustic Points are prepared by the English druggists, by the addition of two per cent. of some adhesive material, which, while not interfering either with the efficacy of the caustic, nor its solubility, renders the nitrate perfectly tough, and as readily cut and pointed as an ordinary slate pencil. They are cast in small conical points, an inch long, and of the diameter of an ordinary lead pencil.

A Medical Sharpshooter.—The *Lancet* says that Dr. Burke Ryan, the surgeon to a rifle regiment, has greatly distinguished himself in rifle shooting, and has been awarded some prizes. The habitual steadiness of practicing surgeons, with their accustomed consentaneous action of eye and hand, should make them good marksmen.

The *Reporter* remarks, that military surgeons, although technically non-combatants, are sometimes obliged to act on the defensive, and a little practice with weapons would be advantageous to them.

Absence of the Uterus.—Dr. R. N. Nelson (*Am. Med. Monthly*), reports three cases of absence of the womb in one family of five sisters, in his own practice. They are described as women of more than ordinary personal attractions —indeed the Dr. enters into the details of their appearance *con amore.* All three have been, or are married, the two elder ones twice, and live quite happily in their marital relations. Of the remaining two, one is married and a mother, and the youngest of the five, who is still unmarried, menstruates regularly, and, to use the words of the writer, "is without defect." The three, without uteri, differ otherwise only from the others in being sparer in flesh, with thin lips and small mammæ, while the latter incline to *embonpoint.* The cases are interesting, as all occurring in one family.

"Onward!"—One of the little pellet-men down in Missouri, knowing our fondness for the humbug, has sent us his valedictory address, recently delivered, and closing with a plaintive appeal to the graduating class to "shout *onward!* throughout the troublesome paths of a weary world." He, himself, shouts "onward!" after this fashion: "But, mark me, gentlemen, if any of you, at any time in your professional career, mistake a dislocation of the inferior maxillary bone for a dynamic disease of the organs, and prescribe your pills, or your powders, your tinctures, triturations, or dilutions, to effect its reduction, your Alma Mater forthwith disowns you, and you deserve no better fate than that your own jaws be dislocated and held so forever, by permanent anchylosis." What a terrible fate is in store for some of them; the homœpathic "Alma Mater" will be orphaned. But then the charitable old creature will still allow them, at least hereaways, to diagnose measles as small-pox, and send their poor, unfortunate patients to the pest-house with well-marked cases of *rubeola.*

CO-EXISTENCE OF DISEASES.—DR. JOHN SWINBURNE, in a letter from the Albany Military Hospital, to which he is attached, to the *Philadelphia Med. and Surg. Reporter*, remarks: "We have received several [patients] with two diseases, as gonorrhœa and rubeola, vaccina and rubeola." Whereupon the *Reporter* interlines the query, "Both in distinct progress at the same time?" And to which we answer, Yankee-fashion, by asking, Why not? Is there anything prophylactic of measles in gonorrhœa, or of vaccina in measles, or *vice versa*, and if not, why should they not be "in distinct progress at the same time?" Dr. Bulkley wrote, in 1851, "the co-existence of two febrile exanthemata in the same individual has now been observed in so many instances, and so many cases of it are now on record, that its occurrence may be considered as beyond doubt." And in the appendix (*C*) to his edition of *Gregory on Eruptive Fevers*, he cites numerous cases in proof, recorded by such men as Drs. John Watson, Tracy, Withering, and Messrs. Gilder, Little, Barnes, Marson, Barthez and Rilliet and Levy; and in closing, remarks, "we could add still further proof that the febrile exanthemata do exist together in the same individual, and run their course simultaneously, but feel that the point is too well established to call for any more extended notice of the subject."

CONSERVATIVE SURGERY.—A correspondent of the *Dublin Medical Press* cites the following instance of the *vis medicatrix naturæ* as unique, as, if true, it undoubtedly is. The union of the smaller bones, such as the phalanges of the fingers and toes, is by no means uncommon, as witness the case reported in the present number of the EXAMINER, and the experience of any surgeon of a dozen years' practice; but the re-union of such large bones as the ulna and radius, when the soft tissues, nerves, vessels, etc., have been divided, and nutrition, apparently, entirely cut off, is certainly remarkable:

In the Life and Opinions of General Sir Charles James Napier, G.C.B., by Lieutenant-General Sir W. Napier, K.C.B., vol. iv, Sir Charles says: Hunter, (General,) who is here, told me a curious thing. Showing me a large sword, which cut off his arm at Burthpoor when leading the assault, he said, that on the rampart a giant, in complete armor, whirling this sword, met him. Hunter held his sword up in defence, but to use his own words, the giant sent it with a *whirr* into the air. Hunter then held up the scabbard, but the blow went through it and his arm, just below the elbow, leaving merely *a bit of skin uncut.* He fell sitting, and held his severed arm in his right hand, while an officer tied a sash above the wound to stop the hæmorrhage: then a surgeon came up, put the *two ends together* and tied them, *and they united.*

Chlorate of Potash in Variola.—Dr. J. T. Reed communicates to the *American Medical Times*, his experience in the use of chlorate of potash in small-pox, with which he treated eighteen out of twenty-two cases, five being of the confluent type, during the months of March and April, last. The advantages claimed are an entire absence of the dyspnœa during the suppurative stage, so constant and troublesome a symptom in variola, "and which was so common with the others, treated with acet. ammon., Rochelle salts, and a Dover's powder, to induce sleep and rest." He says there were some as well-developed cases of the confluent type among those treated with the chlorate, as in those that occurred before he relied exclusively on the salt. "They appeared equally severe, perhaps severer, than any of the former ones. Yet in no instance did they suffer or complain of symptoms of suffocation. What to attribute it to, other than the remedy,—chlorate of potash, freely used, —I know not."

During the last epidemic of small-pox in this city, in the winter of 1857, we were accustomed to exhibit the chlorate in all cases that assumed a typhoid condition, and with markedly beneficial results. But the object sought after then, was a more thorough oxygenation of the blood, and, indirectly, a stimulus to the nervous system, through this natural agency. For the same reason, we consider it applicable in all the eruptive fevers, where there is any evidence of an imperfect arterialization of the blood,—as shown by lividity of the lips and complexion, coldness of the extremities, and sense of oppression in the præcordial region. We seldom treat a case of scarlet fever, now, without using this remedy; and we have been led to infer from its uniformly good effects, that it may possess some specific action in counteracting the blood-poison, or whatever morbific agent it is, which determines the character of the eruptive fevers,—either by the more thorough oxygenization of the blood, or in some unknown mode.

Regeneration of Nerve-Fibre.—Prof. Dalton's experiments on the reproduction of exsected nerves, acquires considerable importance in view of the radical cure for neuralgia so favorably spoken of at the present time. In one of his experiments, a half-inch of the facial nerve of a cat was re-produced in three and a-half months. Bidder, in Germany, and Valentine, in Switzerland, assert that a nerve may be regenerated if extirpated to the extent of three-fourths of, or even an entire inch. In Prof. D.'s lectures on the physiology of the cranial nerves, recently published in the *Am. Med. Times*, he, also, cites experiments, proving, not only that corresponding nerve fibres may be made to unite with each other, *e. g.*, the filaments of the upper branch of the brachial plexus in a cock, with those of the lower branch, and *vice versa*, as M. Flourens had caused, but that filaments of a purely sensitive nerve could be made to unite with those of a purely motor nerve, anatomically, at least, if not physiologically. The experiments proving this last, were made by Messrs. Gluge and Thiernesse, and are interesting enough to warrant us in appending them: They operated upon two of the cranial nerves, one almost exclusively sensitive, the other almost exclusively motor, viz: the lingual branch of the fifth pair, and the hypoglossal. A wound was made in the side of the neck, and the lingual and hypoglossal nerves were exposed and divided. Then the extremity of the lower part of the hypoglossal was simply united with the extremity of the upper part of the lingual, and the wound was closed. But upon examining the animal afterward, it was found that in this case all four extremities of the divided nerves had united together into a single cicatrix; so it was impossible to tell which nerve had united with which. In order to avoid this difficulty, the plan was afterward adopted of uniting together, as I have already said, the lower part of the hypoglossal and the upper part of the lingual, and then dissecting out the remaining portions of each nerve, for the full extent of the wound. The two nerves, the lingual and hypoglossal, were thus allowed to remain in contact with each other; and at the end of a certain time it was found that they had united with each other by a firm ribbon-like cord, which contained in many instances nervous filaments completely distinguishable by the microscope. Notwithstanding this union, however, a galvano-electric discharge, passed through the lingual nerve, did not produce any contraction in the muscles of the tongue below, demonstrating the fact that although sensitive and motor filaments might become united anatomically with each other, there was, nevertheless, no physiological relation between them, and no nervous stimulus could be communicated from one to the other. Now, in doing these experiments, various other points were established at the same time. For example, it was found, after exposing the hypoglossal nerve, that on grasping it with the forceps, certain signs of pain were produced. Now, we know that this is exclusively a moter nerve at its origin, but there are certain sensitive filaments which afterward enter into its composition, and which are due to inosculation with other cranial nerves. In the same manner the facial nerve, which is itself exclusively motor, receives sensitive filaments from certain branches of the fifth pair. The converse is true with regard to the lingual branch of the fifth pair; for although sensitive at its origin, it is joined in its course by a fine bundle of motor filaments, constituting the *chorda tympani.* Thus both these nerves, although exclusively motor or exclusively sensitive at their origins, become mixed nerves before they reach their final distribution.

Cannabis Indica.—Dr. J. R. Reynolds, of London, gives a very valuable paper on "Some of the Therapeutical Uses of Indian Hemp," in the *Archives of Medicine*, in which he asserts that the uncertainty of its action is due to the disregard of one of the following conditions: the choice of a proper case, of a pure article, and the exhibition of a proper dose. Its qualities are soporific, anodyne, and anti-spasmodic; it relieves pain, and spasm, and conduces to sleep; in doing either of these it usually promotes diaphoresis and diuresis; whereas it does not leave behind it headache or vertigo; nor does it affect the appetite nor confine the bowels.

Its beneficial effects are illustrated, 1. In cases of mental or emotional disturbance. 2. For the relief of certain kinds of pain. 3. In certain forms of convulsions. On the other hand, it was absolutely useless in most cases of epilepsy, hypochondria, and the various hysterical affections. To give a bird's eye view of the whole subject, the remedy was, for the *relief of*

EMOTIONAL DISTURBANCES.

*Successful in*—1. Deranged cerebral circulation, with pain and delirium. 2. Incipient insanity after yellow fever. 3. Senile ramollissement.

*Unsuccessful in*—1. Hypochondriasis. 2. Temporary, recurrent religious melancholy. 3. Insomnia with diabetes.

PAINFUL AFFECTIONS.

*Successful in*—1. Nervous irritation from carious teeth. 2. Probable tumor of brain. 3. Probable thickening of spinal meninges. 4. Hemorrhage at roots of 8th and 9th nerves. 5. Syphilitic meningitis. 6. Hemicrania.

*Unsuccessful in*—1. Sciatica. 2. Hysterical hip-joint. 3. Hysterical headache.

AFFECTIONS OF MOTILITY.

*Successful in*—1. Meningitis. 2. Intense cerebral congestion. 3. Obstinate nervous vomiting. 4. Recurrent convulsions.

*Unsuccessful in*—1. Epilepsy.

It does not, like opium, purchase present relief at the expense of future misery. The value of the medicine seems enhanced, because the limitation of its action will enable us to apply it with scientific selection.

A Holland physician, FRONMÜLLER, has also lately produced a treatise on the Indian Hemp, which article he has made an especial study during the past ten years; and his treatise is based upon the clinical observation of no less than a thousand cases of its use We have space only for a summary of the conclusions at which Dr. FRONMÜLLER arrives, which we copy from the *Amer. Jour. of the Med. Sciences*, which in turn reprints from the *B. and F. Med. Chir. Rev.* 1861, from *Vierteljahrschrift für die practische Hielkunde*, 1860. As the result of his observations FRONMÜLLER asserts: 1. That Indian hemp, among all the known medicines which cause stupefaction, is that which produces a narcotism most completely supplying the want of natural sleep, without occasioning any great excitement of the vascular system, without special stoppage of the secretions, without the supervention of unfavorable consequences, and without subsequent paralysis. 2. That Indian hemp, on the other hand, is not so strong nor so certain in its operation as opium. 3. That Indian hemp may be given in all acute inflammatory diseases and in typhus fever. 4. That it is worth a trial to alternate the Indian hemp with opium, in cases where the latter fails. 5. That the best mode of administration is the alcoholic extract, in small pills which contain an addition of the powder of Indian hemp. The lowest dose for producing sleep may be estimated as eight grains given in pills of one grain each.

The Editor of the *Journal* adds a caution about the use of such large doses, where the extract is of the best quality; and we opine it will be found safer to adhere to the customary mode of administration, commencing with small doses and gradually increasing.

### Special Notices.

SUMMER TERM AT THE LIND.—*One recitation and one familiar explanatory lecture daily until the first Monday in October, on the following branches: Materia Medica and Practical Medicine, Prof. J. H. Hollister; Anatomy and Surgery, Prof. R. N. Isham; Chemistry, Prof. F. Mahla; Obstetrics and Diseases of Women, Prof. H. A. Johnson; Clinical Surgery, Prof. E. Andrews; Clinical Medicine, Prof. N. S. Davis.*

*Cliniques at Mercy Hospital, from* 9 *to* 10 *A. M., in the Medical Wards, service of* Prof. DAVIS, *on Tuesdays and Fridays; in the Surgical Wards, service of* Prof. ANDREWS, *on Mondays and Thursdays. At Marine Hospital, from* 9 *to* 10 *A. M.*, *service of* Prof. ISHAM, *on Wednesdays and Saturdays.*

*At Chicago Dispensary, from* 2 *to* 3 *P. M., Surgical Cliniques, service of* Prof. ANDREWS, *on Wednesdays; Diseases of Women and Children, service of* Prof. BYFORD, *on Saturdays.*

*Lectures on Military Surgery, by* Prof. ANDREWS, *in the Amphitheatre of the College, on Wednesday and Saturday afternoons, from* 5 *to* 6.

---

THE DISPENSARY *of the Chicago Charitable Eye and Ear Infirmary, in Ewing's Block, corner of North Clark and North Water Streets, is open daily, from* 11½ *to* 1 *o'clock, for the gratuitous treatment of the poor, afflicted with diseases of the Eye or Ear.* DRS. E. L. HOLMES *and* E. POWELL, *attending Surgeons;* Profs. D. BRAINARD, M. D., *and* J. W. FREER, M. D., *Consulting Surgeons.*

---

THE CHICAGO DISPENSARY, *in Lind's Block, on Market St., near Lake, is open every afternoon from* 2 *to* 3 *o'clock, for the gratuitous treatment of the indigent sick.* Profs. W. H. BYFORD, M. D., *and* E. ANDREWS, M. D., *Physicians and Surgeons in charge.*

